# A pan-cancer analysis reveals CHD1L as a prognostic and immunological biomarker in several human cancers

**DOI:** 10.3389/fmolb.2023.1017148

**Published:** 2023-03-23

**Authors:** Mohamed A. Soltan, Muhammad Alaa Eldeen, Refaat A. Eid, Najiah M. Alyamani, Leena S. Alqahtani, Sarah Albogami, Ibrahim Jafri, Moon Nyeo Park, Ghadi Alsharif, Eman Fayad, Gamal Mohamed, Rihab Osman, Bonglee Kim, Mohamed Samir A. Zaki

**Affiliations:** ^1^ Department of Microbiology and Immunology, Faculty of Pharmacy, Sinai University, Ismailia, Egypt; ^2^ Cell Biology, Histology and Genetics Division, Zoology Department, Faculty of Science, Zagazig University, Zagazig, Egypt; ^3^ Pathology Department, College of Medicine, King Khalid University, Abha, Saudi Arabia; ^4^ Department of Biology, College of Science, University of Jeddah, Jeddah, Saudi Arabia; ^5^ Department of Biochemistry, College of Science, University of Jeddah, Jeddah, Saudi Arabia; ^6^ Department of Biotechnology, College of Sciences, Taif University, Taif, Saudi Arabia; ^7^ Department of Pathology, College of Korean Medicine, Kyung Hee University, Seoul, South Korea; ^8^ College of Clinical Laboratory Sciences, King Saud bin Abdulaziz University for Health Sciences, Jeddah, Saudi Arabia; ^9^ Department of Human Anatomy, Jazan University, Jazan, Kingdom of Saydi Arabia; ^10^ Department of Anatomy, College of Medicine, King Khalid University, Abha, Saudi Arabia; ^11^ Anatomy Department, College of Medicine, King Khalid University, Abha, Saudi Arabia; ^12^ Department of Histology and Cell Biology, College of Medicine, Zagazig University, Zagazig, Egypt

**Keywords:** CHD1L, pan-cancer, differential expression, prognosis, tumor immunotherapy, biomarker

## Abstract

**Introduction:** Several recent studies pointed out that chromodomain-helicase-DNA-binding protein 1-like (CHD1L) is a putative oncogene in many human tumors. However, up to date, there is no pan-cancer analysis performed to study the different aspects of this gene expression and behavior in tumor tissues.

**Methods:** Here, we applied several bioinformatics tools to make a comprehensive analysis for CHD1L. Firstly we assessed the expression of CHD1L in several types of human tumors and tried to correlate that with the stage and grade of the analyzed tumors. Following that, we performed a survival analysis to study the correlation between CHD1L upregulation in tumors and the clinical outcome. Additionally, we investigated the mutation forms, the correlation with several immune cell infiltration, and the potential molecular mechanisms of CHD1L in the tumor tissue.

**Result and discussion:** The results demonstrated that CHD1L is a highly expressed gene across several types of tumors and that was correlated with a poor prognosis for most cancer patients. Moreover, it was found that CHD1L affects the tumor immune microenvironment by influencing the infiltration level of several immune cells. Collectively, the current study provides a comprehensive overview of the oncogenic roles of CHD1L where our results nominate CHD1L as a potential prognostic biomarker and target for antitumor therapy development.

## 1 Introduction

The process of tumorigenesis was proved to be a complex one involving a series of interactions between various genes that consequently transfers the cells from the normal state to the cancerous condition (Gharib et al., 2021; Mahaboob Batcha et al., 2022). Hence, deep studying of different oncogenes is an essential process to explore the molecular mechanisms of different genes in cancer development (Hnisz et al., 2016). A pan-cancer analysis that employs available databases such as The Cancer Genome Atlas (TCGA) gave us a great opportunity to analyze the expression and the behavior of a specific gene in a large list of tumors in an economic and time-saving approach with the currently developed bioinformatics tools (Tomczak et al., 2015; Blum et al., 2018).

Human *CHD1L* was firstly identified more than 10 years ago by Ma et al. (2008). The exact genomic location of *CHD1L* is at Chr 1q21.1 between the flavin-containing monooxygenase 5 (*FMO5*) gene and prostaglandin reductase pseudogene (LOC100130018) where its length is 53,152 base pairs. Investigation of the CHD1L protein sequence revealed that it belongs to the SNF2-like family and has two domains SNF2_N and a Macro domain where the former domain contains 280 amino acids, and its sequence homology with another SNF2-like family member, chromodomain helicase DNA binding protein 1 (CHD1) generated 45% identity (Cheng et al., 2013) and because of this similarity, *CHD1L* has given its name. The basic functions of CHD1L protein were estimated by relying on the structural similarity with CHD1. CHD1 binds with the cellular DNA and organizes ATP-dependent nucleosome assembly and modifies the chromatin structure (Ahel et al., 2009). Consequently, CHD1L was predicted to have a significant role in cellular DNA repair and chromosome integrity maintenance. It is noteworthy that CHD1L does not have a chromodomain like CHD1 protein. Instead, it contains a macro domain therefore CHD1L is not able to recognize methylated histone tails, and the macro domain gives it a PAR-dependent chromatin remodeling activity and consequently aids in the cellular DNA repair mechanisms within a chromatin context (Gottschalk et al., 2009).

Amplification of the 1q21 region, where the *CHD1L* gene belongs, was noticed in several solid tumors (Kwong et al., 2004; Niini et al., 2010; Ji et al., 2013). Specific investigation of the CHD1L roles in cell proliferation, migration, and apoptosis has revealed its potential role in tumorigenesis (Chen M. et al., 2009). It was reported that CHD1L enhanced cell motility and induced filopodia generation through ARHGEF9-mediated Cdc42 activation, a process that collectively stimulated tumor cell migration, invasion, and metastasis (Chen et al., 2010). Additionally, SPOCK1 was reported to be upregulated by the action of CHD1L in HCC and consequently, cells experienced apoptosis inhibition through the induction of the Akt signaling pathway. Moreover, HCC cells with a high rate of SPOCK1 expression were noticed to be more invasive in mice than the ones with normal SPOCK1 expression (Chen et al., 2010). Moving to another type of tumor, CHD1L was revealed to be a stimulating factor for breast cancer progression through the MDM2/p53 signaling pathway (Wang et al., 2019). Furthermore, CHD1L was involved in pancreatic cancer proliferation through the activation of the Wnt/β-catenin pathway (Liu C. et al., 2017).

Although it has been established that CHD1L plays an important role in the progression of different types of tumors, there is a lack of studies that analyze the collective action of CHD1L in a group of tumors, and for this purpose, we present here the first systematic pan-cancer analysis of CHD1L. The current study analyzes the expression profile of CHD1L across several types of tumors and tried to correlate that with the prognosis and the infiltration of the immune cells. We also investigated *CHD1L* gene mutation types besides the estimation of the interacted and correlated gene network. This comprehensive study demonstrates the predicted molecular roles of CHD1L in several cancer types in addition to its influence on clinical prognosis.

## 2 Materials and methods

### 2.1 Gene expression analysis

Firstly, the level of CHD1L gene expression in several tumors versus normal tissue was visualized through the data on the Tumor Immune Estimation Resource, version 2 (TIMER2.0) (Li et al., 2020). After analysis on TIMER2.0, it was found that there are some tumors without normal tissue for comparison, consequently, the Gene Expression Profiling Interactive Analysis (GEPIA) database (http://gepia.cancer-pku.cn/index.html) was utilized to compare CHD1L gene expression in normal and cancerous tissue in more tumor types (Tang et al., 2017). In order to investigate the relationship between CHD1L gene expression and the grade of the tumor, we employed the TISBID website (Ru et al., 2019), while Human Protein Atlas (HPA) database (https://www.proteinatlas.org/) (Sjöstedt et al., 2020) was explored to get an overview of CHD1L protein level in various tumor types. Finally, TNMplot (differential gene expression analysis in Tumor, Normal and Metastatic tissues) online server was used with its Kruskal–Wallis test for significance assay to assess CHD1L expression in tumor, normal, and metastatic tissues (Bartha and Győrffy, 2021).

### 2.2 Protein expression and immunohistochemistry (IHC) staining

In order to investigate the differential expression of CHD1L protein between normal and cancerous samples UALCAN tool, which performs protein expression analysis based on the data from the Clinical Proteomic Tumor Analysis Consortium (CPTAC), was employed (Chandrashekar et al., 2017). Moreover, we employed HPA to track the IHC images of CHD1L expression in normal and cancerous tissues, for the tumors that showed a significant difference in UALCAN analysis, to confirm our results.

### 2.3 Survival prognosis analysis

Firstly, the GEPIA2.0 webserver was employed to assess patient survival where we tried *CHD1L* in the “survival analysis” section, selected the full tumor list in the TCGA cohort, and obtained the heatmap for the two available methods on the server (overall survival and disease-free survival). Following that, we utilized the KM plotter (Lánczky and Győrffy, 2021) to analyze the correlation between CHD1L expression and patients’ survival in five cancer models (breast, ovarian, lung, gastric, and liver cancer).

### 2.4 Gene alteration analysis

We used the cBioPortal web server to perform a comprehensive analysis for CHD1L mutations (Gao et al., 2013). In order to run this analysis, We selected “TCGA PanCancer Atlas Studies” to be the source for our analysis where we tracked four major points. Firstly, the output of the alteration frequency and mutation type was obtained from the “cancer types summary” tab. Secondly, “Plots” tab data was investigated to acquire gene mutation frequency. Thirdly, the “Mutations” tab was accessed to visualize CHD1L mutation sits. Finally, we accessed the “Comparison/Survival” tab to study the correlation between CHD1L mutations and patients’ survival.

### 2.5 CHD1L methylation analysis

Cellular epigenetic regulation is an essential mechanism to control gene expression patterns (Qu et al., 2013), where DNA methylation represents a major component of this regulatory machinery (Tran et al., 2021). In the current study, we accessed two web servers, namely, UALCAN (Chandrashekar et al., 2022) and SMART app (Li et al., 2019a) to perform the methylation analysis for the *CHD1L* gene where the former server was used to obtain the promoter methylation levels from the TCGA dataset with sample size more than 20, and the later one was accessed to get CpG-aggregated methylation data.

### 2.6 Immune reactivity assessment

Firstly, we used the TIMER2 web server (Li et al., 2016) to find the relation between CHD1L expression and immune cells, that may perform positive or negative roles on tumor progression, across the panel of TCGA tumors. We introduced our target gene name in the “gene” module under the “immune” partition and selected two types of infiltrating immune cells, namely, myeloid-derived suppressor cells (MDSCs) and natural killer T cells (NKT), with opposing roles in tumor development (Umansky et al., 2016; Bae et al., 2019) and then we obtained heatmap and scatter plots describing our studied correlation. Following that, we accessed SangerBox online server where we explored the correlation between our target gene and three variables, namely, immune checkpoints, microsatellite instability (MSI), and the tumor mutational burden (TMB).

### 2.7 Protein-protein interaction and enrichment analysis

In order to study the proteins that could interact with or correlate to CHD1L, we have accessed two online servers. Firstly, we employed the STRING database (Szklarczyk et al., 2021) to obtain the network of CHD1L interacting proteins. We obtained the top 50 interacting proteins by setting “Experiments” as the active interaction source and “low confidence” as the interaction score. Secondly, we used the GEPIA2 database to obtain the top 100 correlated genes to CHD1L based on the analysis of TCGA tumors where the “Correlation Analysis” module on the same database and “Gene_Corr” module in TIMER were employed to get the correlation curves and the heatmap for the top five correlated genes. Following that, we accessed the online server (http://bioinformatics.psb.ugent.be/webtools/Venn/) to find the common proteins in both “CHD1L interacting” and “CHD1L correlating” lists. Finally, after combining both lists and removing the duplicates, the generated data set was submitted to the Database for Annotation, Visualization, and Integrated Discovery (DAVID) (Sherman et al., 2022) to perform a functional enrichment analysis where the results were visualized using “ggplot2” package of R (4.2.0).

## 3 Results

The abbreviations and the full name of analyzed tumors in the current study are shown in supplementary table 1.

### 3.1 CHD1L increased expression in many tumor types

The current study firstly investigated the distribution of CHD1L in normal tissue. Regarding the cellular level, CHD1L was mainly found in the nucleus (Supplementary Figure 1A), moving to the tissue level, the data from Human Protein Atlas (HPA) showed that CHD1L RNA is mainly found in the brain, liver, bone marrow, and lymphoid tissues while CHD1L protein is expressed in several tissues (Supplementary Figure 1B).

**FIGURE 1:**
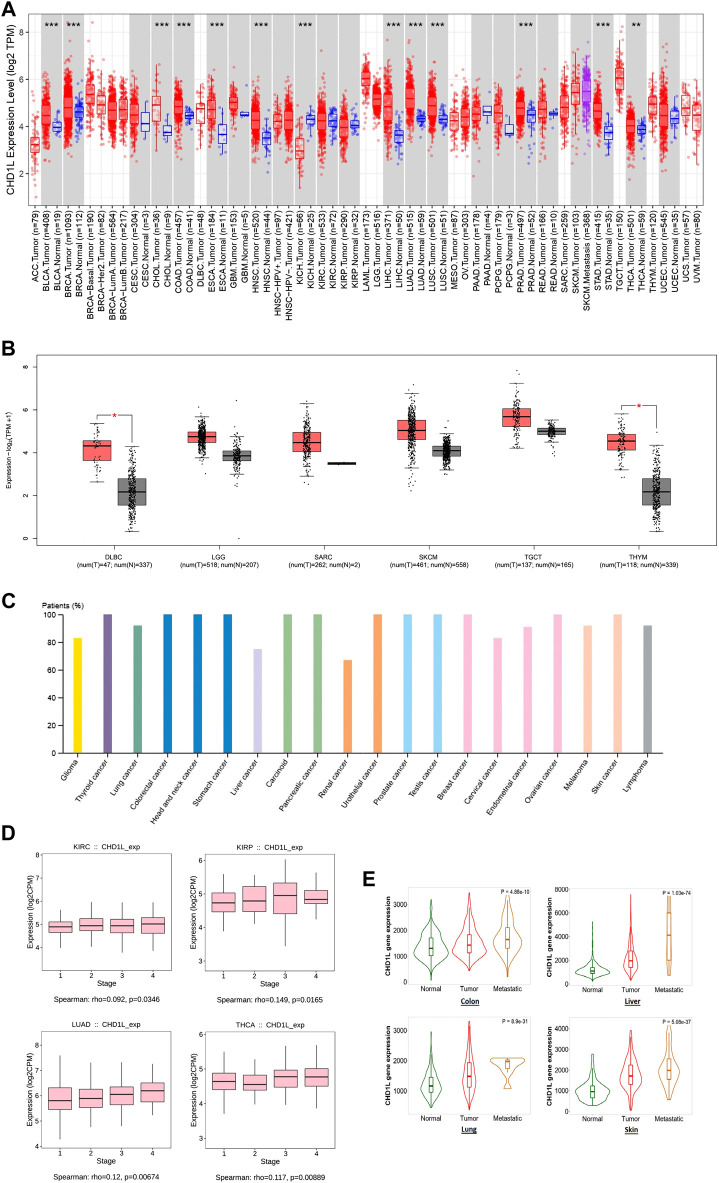
CHD1L expression assessment in human cancers. **(A)** Differential expression of CHD1L in a panel of TCGA tumors analyzed by TIMER2.0. **(B)** The tumors that lack normal tissue for comparison in TIMER2.0. database and experienced a trend of elevated CHD1L expression in tumor versus normal tissue when analyzed in the GEPIA database. **(C)** High level of CHD1L expression in several human cancers analyzed on HPA. **(D)** Tumors experienced a positive correlation between CHD1L expression and the tumor stage when analyzed with the TISDIB webserver. **(E)** Tumors experienced a consistent positive correlation between CHD1L expression and tissue type (normal-tumor-metastatic).(a),(b)

Secondly, TIMER2 was utilized to investigate the differential expression of our targeted gene between cancerous and adjacent normal tissue. It was found that CHD1L is significantly upregulated in many tumors (Figure 1A) such as BLCA, BRCA, CHOL, COAD, ESCA, HNSC, LIHC, LUAD, LUSC, PRAD, STAD (*p* < 0.001) and THCA (*p* < 0.01). Due to the absence of normal tissue expression to be used for comparison for some tumors in TIMER2, we obtained the differential expression in normal and cancerous tissues for these tumors from Gene Expression Profiling Interactive Analysis (GEPIA) database. A trend of an increased expression of CHD1L in tumor versus normal tissue was found in LGG, SARC, SKCM, TGCT, DLBC, and THYM (the last two were statistically significant, *p* < 0.05) (Figure 1B), while ACC, LAML, OV, and UCS experienced little upregulation (with no statistical significance) in normal versus tumor tissues (Supplementary Figure 2). Concurrently, The HPA was investigated to assess CHD1L protein expression in cancerous patients and the results indicated that CHD1L protein was overexpressed in most human cancers (Figure 1C) where it was overexpressed in about 100% of thyroid, colorectal, head and neck, stomach, carcinoid, pancreatic urothelial, prostate, testis, breast, ovarian and skin cancer patients.

After analyzing the differential expression of CHD1L in normal and cancerous tissue, we aimed to explore the relationship between CHD1L expression and the cancer stage. For this purpose, we employed the TISDIB webserver where the generated data demonstrated that the expression of CHD1L was positively correlated with the tumor stage of LUAD and THCA (*p* < 0.01), KIRP and KIRC (*p* < 0.05) (Figure 1D). On the other hand, other analyzed tumors showed no significant correlation (Supplementary Figure 3) and STAD showed a negative correlation between CHD1L expression and tumor stage (*p* < 0.05) (Supplementary Figure 4). Finally, we applied the “compare tumor, normal, and metastasis” module of the TNMplot web server to associate the CHD1L mRNA expression level with the metastasis. The generated data revealed that, in colon, liver, lung, and skin cancers, CHD1L showed a significantly upregulated expression when we set a comparison between normal and tumor tissues and kept this trend in the metastatic versus the tumor tissues (Figure 1E). This trend was disturbed in other analyzed tumors such as the beast, intestine, and kidney cancers (Supplementary Figure 5).

### 3.2 Differential protein expression

**FIGURE 2:**
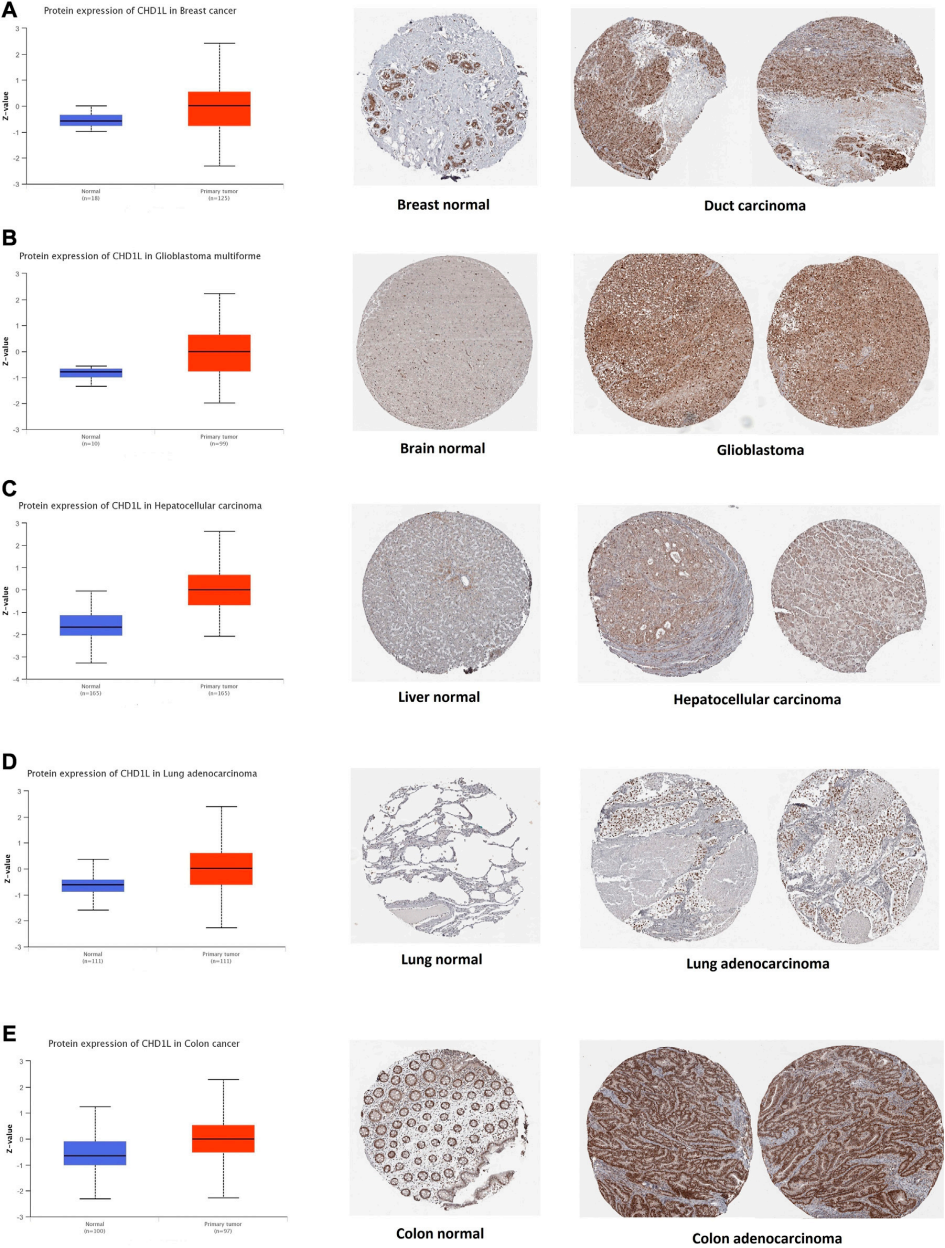
Tumors experienced a statistically significant higher CHD1L protein expression in the tumor sample versus normal one (left side) and IHC staining for normal tissue (middle) and cancerous one (left) demonstrated the same results. **(A)** Breast. **(B)** Brain. **(C)** Liver. **(D)** Lung. **(E)** Colon.

After the analysis of CHD1L on its transcriptional level, we assessed its protein level through the usage of the large-scale proteome data available by the National Cancer Institute`s CPTAC dataset. The results demonstrated that CHD1L protein expression was significantly upregulated in LUAD, LIHC, glioblastoma multiforme, colon, and breast cancer tumor tissues in a comparison with a normal one (Figures 2A–E, *p* < 0.001). Following that we obtained the IHC figures for the normal and cancerous tissues to confirm our previous findings and the results were matching as the staining was low to intermediate in the normal tissue of the breast, brain, liver, lung, and colon while it was intermediate to high in the corresponding cancerous one (Figures 2A–E).

### 3.3 Increased CHD1L level estimates poor clinical outcomes

**FIGURE 3:**
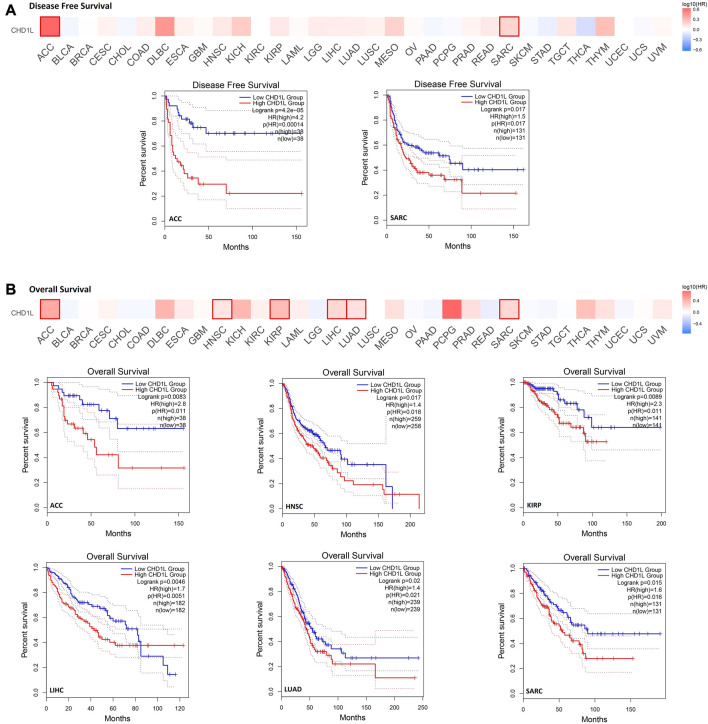
The correlation between CHD1L expression and the clinical outcome. **(A)** disease-free survival **(B)** overall survival as assessed from the GEPIA database.

In order to analyze the correlation between CHD1L expression and patients’ survival, we used two databases, namely, GEPIA and Kaplan- Meier (KM) plotter. From the GEPIA database, we found that the expression of our target gene is linked to a poor prognosis for ACC (*p* < 0.001) and SARC (*p* < 0.05) in terms of disease-free survival (DFS) (Figure 3A). On the other hand, analysis of patients’ overall survival (OS) demonstrated that not only ACC (*p* < 0.01) and SARC (*p* < 0.05) patients assessed with poor prognosis, but also HNSC (*p* < 0.05), KIRP (*p* < 0.01), LIHC (*p* < 0.01), and LUAD (*p* < 0.05) patients behave similarly (Figure 3B).

**FIGURE 4:**
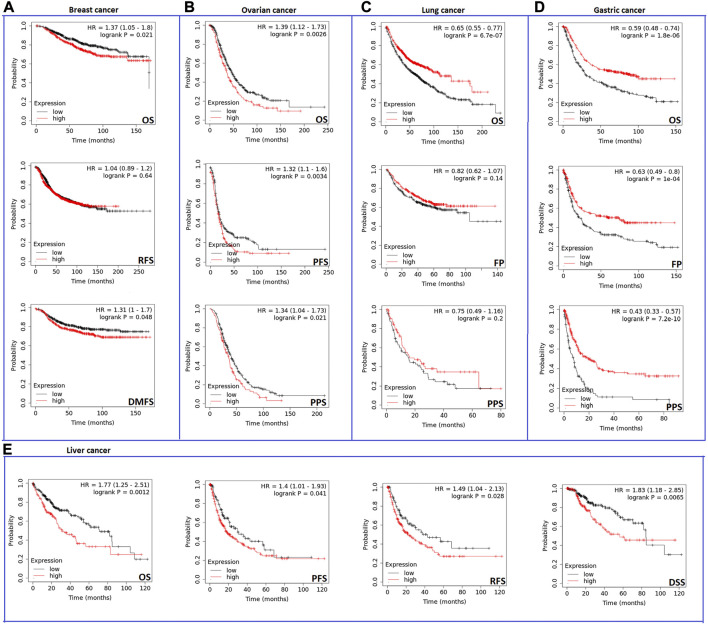
The correlation between CHD1L expression and the survival prognosis as assessed with Kaplan–the Meier plotter tool for **(A)** Breast, **(B)** Ovarian, **(C)** Lung, **(D)** Gastric, and **(E)** Liver cancer.

Analysis results from the second server showed that the expression of CHD1L influenced negatively OS and distant metastasis-free survival (DMFS) (*p* < 0.05) (Figure 4A) but not relapse-free survival (RFS) in breast cancer patients. Regarding ovarian cancer, CHD1L was predicted to correlate with poor OS (*p* < 0.01), progress-free survival (PFS) (*p* < 0.01), and post-progression survival (PPS) (*p* < 0.05) (Figure 4B). Moreover, our analyzed gene was related to poor OS (*p* < 0.001) in lung cancer (Figure 4C). Gastric cancer represented the most affected cancer type in terms of patients’ survival as the OS, first progression (FP), and PPS were affected with CHD1L expression (*p* < 0.001) (Figure 4D). Finally, liver cancer demonstrated poor OS, disease-specific survival (DSS) (*p* < 0.01), PFS, and RFS (*p* < 0.05) (Figure 4E) with CHD1L expression. The results from both databases indicate that an overall poor clinical outcome is expected with CHD1L expression in cancer patients.

### 3.4 CHD1L mutation analysis

**FIGURE 5:**
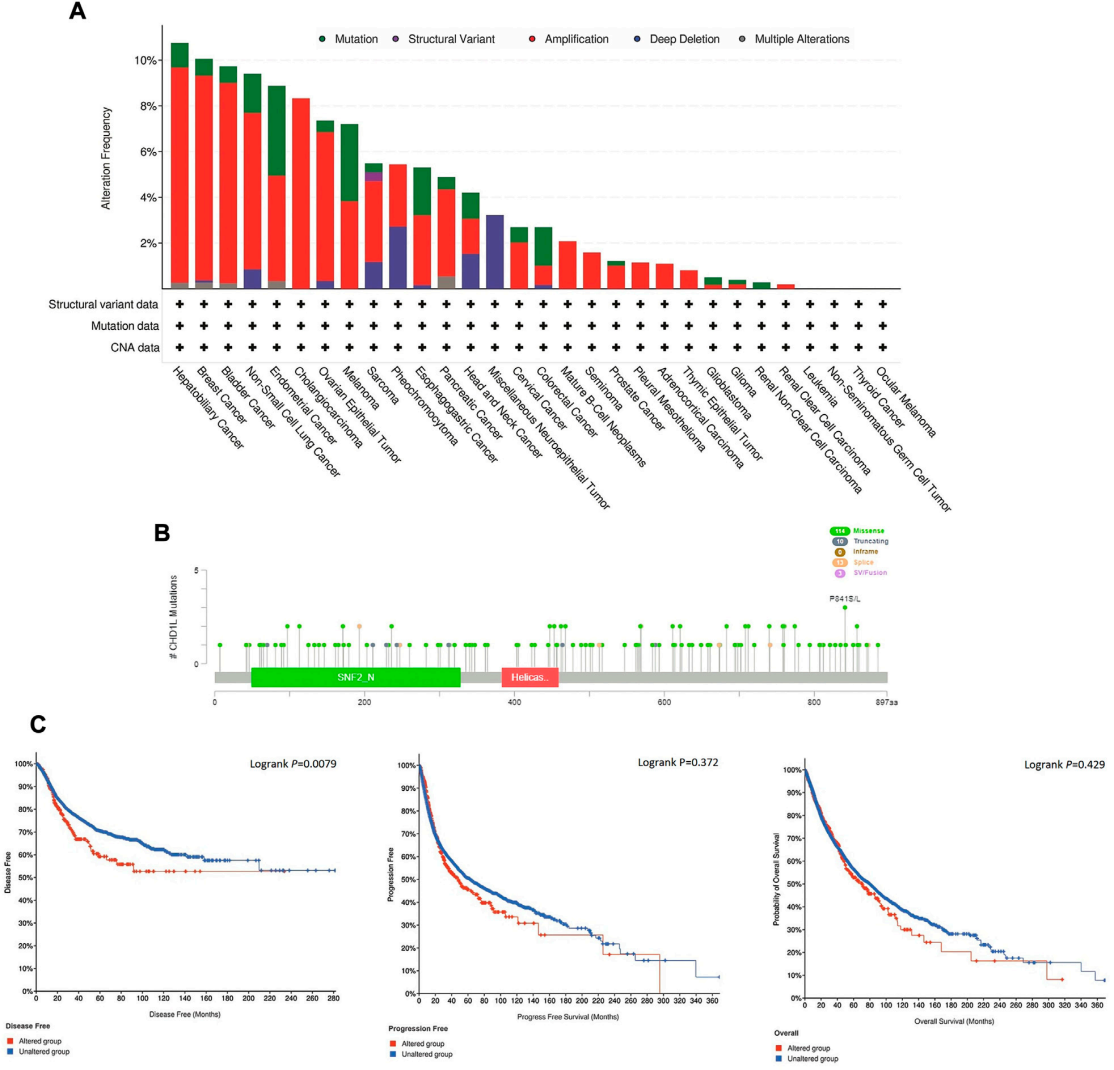
Mutation assessment for CHD1L using the cBioPortal tool. **(A)** The alteration frequency with mutation type in a panel of analyzed human cancers. **(B)** A map representation for sites and types of CHD1L mutations. **(C)** Assessment of the correlation between CHD1L mutation and disease-free, progression-free, and overall survival.

Here, we explored the copy number alteration (CNA) of CHD1L in human samples. According to the output from the cBioPortal web server, the most frequent CHD1L alteration was found in hepatobiliary cancer with an alteration frequency of more than 10%, where amplification represented the dominant alteration form in this tumor. It is noteworthy that most of the analyzed tumors experienced “amplification” as the most common form of genetic alteration except for neuroepithelial tumor, which showed deep deletion as the major shape of alteration, and head and neck cancer which showed three forms of alteration (namely, mutation, amplification, and deep deletion) with a close percentage of occurrence (Figure 5A). Next, we analyzed the count of different mutation forms and it was found that missense mutations were the most frequent form (Supplementary Figure 6). Following that we investigated sites and types of CHD1L mutations (Figure 5B) where we found 140 total mutations with missense mutations in the first place with 114 recorded samples. Regarding gene location, the site P841 S/L was reported to be the most altered site with three missense mutations (2 samples with cutaneous melanoma and one with lung squamous cell carcinoma). Finally, we analyzed the correlation between CHD1L alteration and patients’ survival. In 5,353 total patients, genetic alteration of CHD1L showed poor prognosis in DFS (*p* = 0.0079), but there was no significant difference in PFS (*p* = 0.372, total number of patients = 10,613) or OS (*p* = 0.429, total number of patients = 10,803), compared with patients without CHD1L alterations (Figure 5C).

### 3.5 CHD1L methylation analysis

**FIGURE 6:**
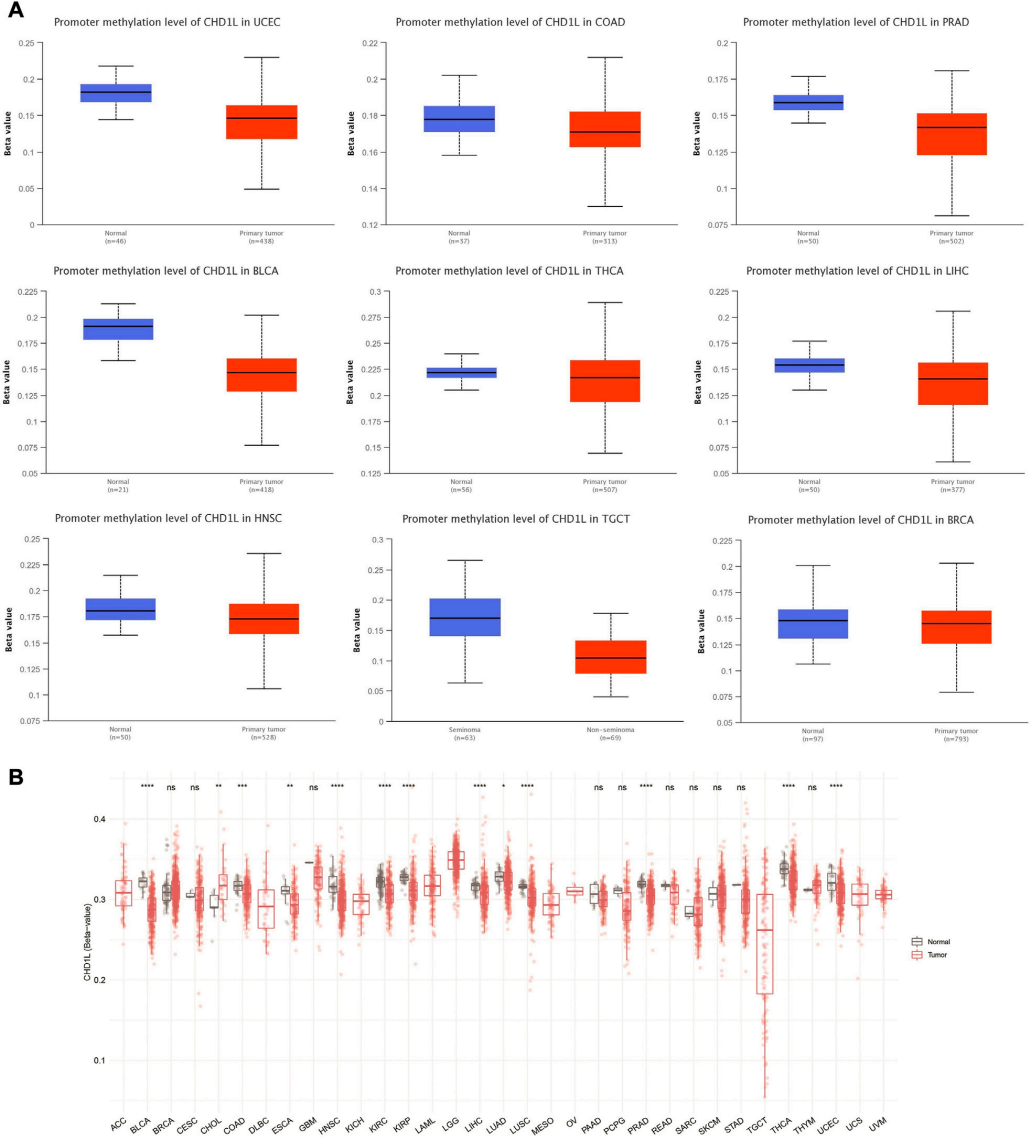
Differential methylation analysis of CHD1L in tumor samples versus normal ones. **(A)** Tumors experienced higher methylation in the CHD1L promoter region in normal samples versus tumors as assessed by UALCAN analysis. **(B)** Analysis of CpG-aggregated methylation of CHD1L in a list of human tumors.

We performed *CHD1L* methylation analysis to study the correlation between CHD1L DNA methylation and tumor progression. Regarding promoter methylation, the output from UALCAN analysis demonstrated that nine tumors, namely, UCEC, COAD, PRAD, BLCA, LIHC, HNSC, TGCT, BRCA (*p* < 0.001), and THCA (*p* < 0.01) experienced higher promoter methylation in normal samples versus tumor ones (Figure 6A). On the other hand, only one tumor, KIRC, experienced lower promoter methylation in normal versus tumor samples (*p* < 0.001) (Supplementary Figure 7B) and four tumors (LUAD, LUSC, KIRP, and PAAD) have shown non-significant difference (Supplementary Figure 7A). A similar pattern was noticed from the results of the SMART app where statistically significant results from all tumors, except CHOL, showed a higher methylation level of CHD1L in normal versus tumor samples (Figure 6B).

### 3.6 CHD1L correlates with immune infiltration in several tumor types

**FIGURE 7:**
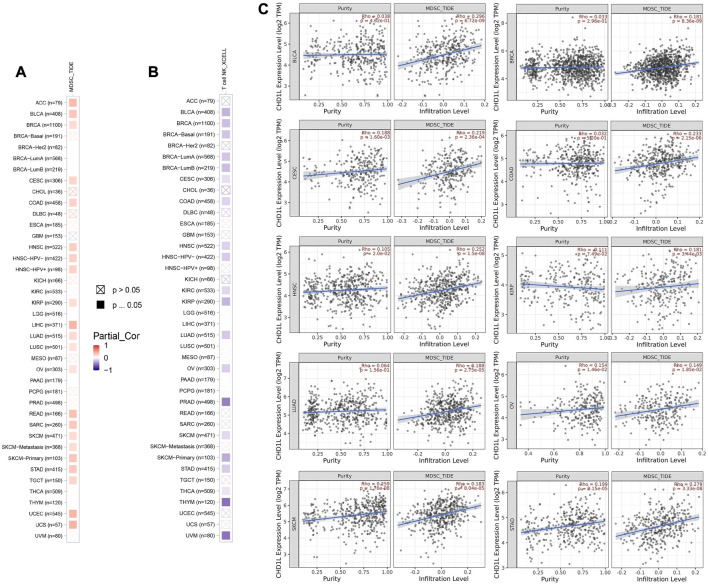
The correlation between CHD1L expression level and infiltration of **(A)** myeloid-derived suppressor cells (MDSC) and **(B)** Natural killer T cells in a panel of human cancers. **(C)** Scatter plots that demonstrate the correlation between the expression of CHD1L and the infiltration level of MDSC.

First of all, we tried to find the correlation between CHD1L expression and the infiltration of two types of immune cells with opposing roles against tumor growth. Analysis of MDSC infiltration, a cell with immunosuppressive roles in tumor (Gabrilovich and Nagaraj, 2009), in the panel of TCGA tumors showed that more than half of the studied tumors experienced a positive correlation between CHD1L expression and MDSC infiltration. It is noteworthy that there was no tumor from the analyzed panel that witnessed a negative correlation between CHD1L expression and MDSC infiltration (Figure 7A). On the other hand, analysis of NKT cell infiltration, a cell that has a strong anti-tumor action and was selected as a target for cancer immunotherapy development (Liu et al., 2021) demonstrated that half of the analyzed tumors experienced a negative correlation between CHD1L expression and NKT cells infiltration (Figure 7B), and again there was no tumor from the analyzed panel witnessed a positive correlation between CHD1L expression and NKT infiltration. After results filtration, we found 10 tumors, namely, BLCA, BRCA, CESC, COAD, HNSC, KIRP, LUAD, OV, SKCM, and STAD, experiencing a positive correlation between CHD1L expression and MDSC in addition to a negative correlation between the same gene expression and NKT cells infiltration. The scatter plots that demonstrate the correlation between the expression of CHD1L and the infiltration level of MDSC in these 10 filtered tumors are shown in (Figure 7C), while that of NKT cells is shown in the Supplementary Figure 8.

**FIGURE 8:**
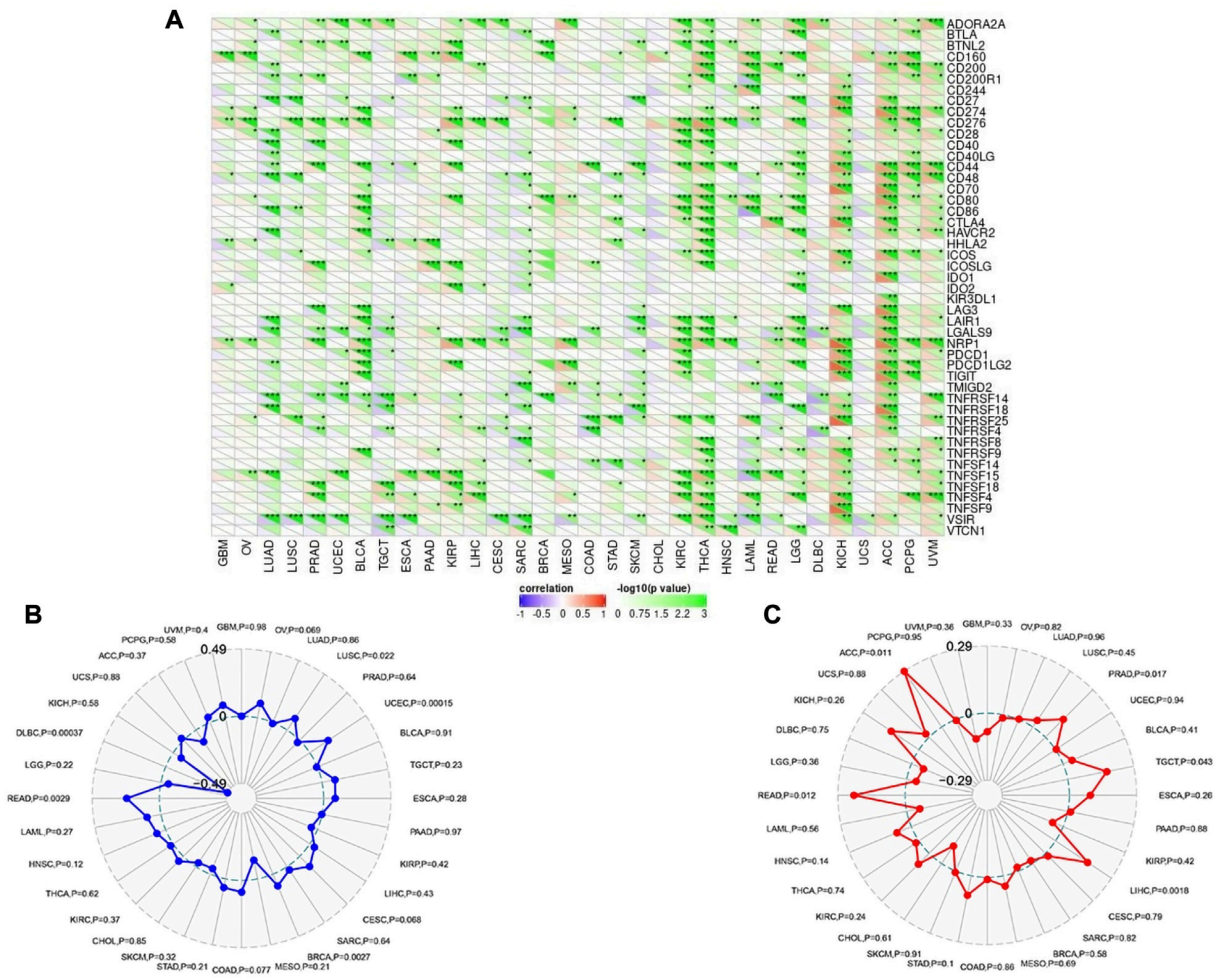
Correlations of CHD1L expression with immune checkpoints, MSI, and TMB. **(A)** Heatmap correlating the immune checkpoints and CHD1L across a list of human tumors. **(B)** and **(C)** are radar charts showing the overlaps of CHD1L with MSI and TMB respectively.

Following that, SangerBox online server was employed to find the correlation of CHD1L expression with immune checkpoint, MSI, and TMB. Regarding immune checkpoint; expression of CHD1L in ACC, KICH, THCA, and KIRC were found to be positively correlated with several immune checkpoint genes while tumors UCS and CHOL experienced no significant correlation between our target gene expression and most of the immune checkpoint genes (Figure 8A). Moving to MSI analysis, the expression of CHD1L was found to be significantly positively correlated with MSI in READ, LUSC, UCEC, and BRCA while only one tumor, DLBC, demonstrated a significant negative correlation between CHD1L expression and MSI (Figure 8B). Finally, an analysis of our target gene expression and TMB showed a significantly positive correlation in ACC, PRAD, TGCT, LIHC, and READ (Figure 8C).

### 3.7 Analysis of interacting and correlated proteins to CHD1L

**FIGURE 9:**
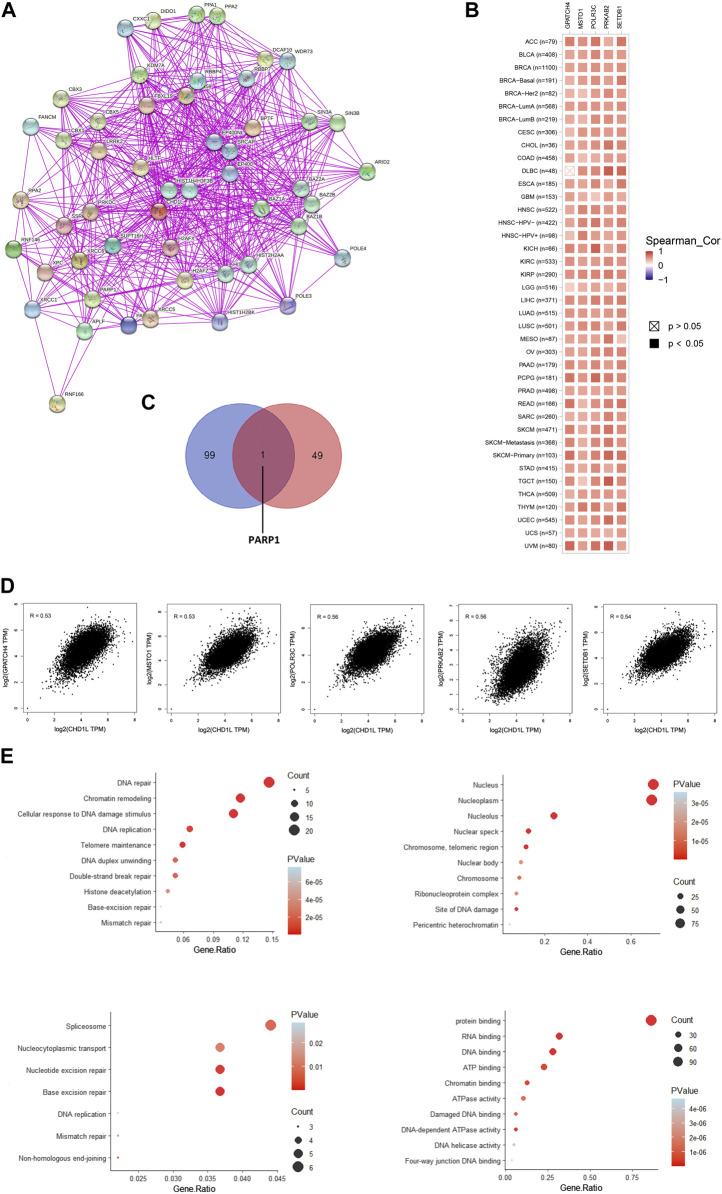
CHD1L-protein network interactions. **(A)** A map of the top 50 CHD1L interacting proteins as determined by the STRING database. **(B)** Heatmap for top five CHD1L-correlated proteins in the tumor tissue. **(C)** Venn diagram showing the intersection between CHD1L interacting and correlating proteins. **(D)** Expression correlation between CHD1L and genes (GPATCH4, MSTO1, POLR3C, PRKAB2, and SETDB1) as determined by GEPIA2. **(E)** KEGG/GO enrichment analysis based on CHD1L-binding and interacted genes.(a), (b)

Based on the above-mentioned results, it is found that CHD1L has a clear association with cancerous patients’ survival and affects the immune cells in the tumor microenvironment. Consequently, it is important to analyze the potential molecular mechanisms of this gene in several tumors. For this purpose, we employed the STRING database to obtain the top 50 experimentally validated CHD1L-interacting proteins where they were presented as a protein-protein interaction network (Figure 9A). Furthermore, the GEPIA2 webserver was explored to get the 100 genes correlated to CHD1L in the panel of TCGA tumors, and the “Correlation Analysis” module was employed to obtain the plots of the top five correlating genes and they were ordered as the following: POLR3C (R = 0.56), PRKAB2 (R = 0.56), SETDB1 (R = 0.54), GPATCH4 (R = 0.53), and MSTO1 (R = 0.53) (Figure 9D). Additionally, a heatmap, generated through the “Gene_Corr” module at TIMER, confirmed the significant positive correlation between those five genes and CHD1L in the full list of TCGA cancers (except for DBLC where the correlation with GPATCH4 was insignificant) (Figure 9B). Following that we compared the above generated two lists and found the gene PARP1 to be the only duplicated one (Figure 9C). Following duplicate removal, a new dataset generated from the combined two lists was submitted to DAVID to run (KEGG) and Gene Ontology (GO) enrichment analyses. The output from biological process analysis revealed that our studied gene list could be related to DNA repair, chromatin remodeling, cellular response to DNA damage, and DNA replication. Regarding cellular components, most of the genes were enriched for the nucleus and nucleoplasm. Additionally, the submitted list of genes was enriched for protein, RNA, and DNA binding when it was analyzed for its molecular function. Finally, the KEGG pathway analysis demonstrated that CHD1L is highly related to spliceosome, nucleocytoplasmic transport, nucleotide and base excision repair (Figure 9E).

## 4 Discussion

CHD1L is an essential cellular protein that was found to be involved in many cellular processes such as chromosome remodeling and integrity maintenance, DNA repair, and controlling the transcriptional status of several genes through the binding with DNA (Xiong et al., 2021). Additionally, many reports have correlated this gene with cell metastasis and tumorigenesis (Liu Z. H. et al., 2017). Analysis of surgical samples from 112 pancreatic cancer patients revealed that the elevation in CHD1L was positively correlated with the patients’ poor survival where the Wnt/β-catenin pathway was linked to the effect of CHD1L on tumor cells proliferation (Liu C. et al., 2017). Studying the oncogenic role of CHD1L showed that it can upregulate two genes, mouse double minute two homolog (MDM2) and methionyl aminopeptidase 2 (METAP2), where the first one was associated with breast cancer progression (Wang et al., 2019) and the later was linked to epithelial ovarian cancer metastasis and invasiveness (He et al., 2020). CHD1L had the ability to bind to the promoter of ZKSCAN3, inhibiting its transcription and stimulating the hepatocellular carcinoma migration (Zhang et al., 2021). Moreover, the same tumor survival was found to be kept by CHD1L through the suppression of nucleus-to-mitochondria translocation of nur77 (Chen L. et al., 2009). CHD1L was also found to be involved in cisplatin resistance in NSCLC through the upregulation of the ABCB1–NF-κB axis (Li et al., 2019b). As a general finding, CHD1L was attributed to poor prognosis and metastasis in other tumors such as gastric (Su et al., 2014), colorectal (Abbott et al., 2020), and bladder cancer (Tian et al., 2013).

Although several studies tried to analyze the oncogenic of CHD1L in several human cancers, there is a lack of a comprehensive study that can deal with the effect of CHD1L from many perspectives in a list of several human tumors. It is already established that the tumor microenvironment is a complex one where several factors are involved in tumor development, immune response against this abnormal growth, patients’ response to tumor therapy, and overall survival (Zabady et al., 2022). This complex status of the tumor confirms the requirement of a deep approach that can correlate a targeted gene with tumor progression through different points of analysis and for this purpose the current study applied a pan-cancer analysis for the oncogenic behavior of CHD1L. We started our study by analyzing the distribution of CHD1L in human tissue where it was found to be expressed in several organs. An important characteristic of the oncogenic proteins is their upregulation in tumor tissue than the normal one and for this reason, our next step was to study the differential expression of CHD1L in a list of human tumors where it was found to be significantly upregulated in BLCA, BRCA, CHOL, COAD, ESCA, HNSC, LIHC, LUAD, LUSC, PRAD, STAD, THCA DLBC, and THYM. Following that, our study tried to reveal if there is a relation between CHD1L expression and the cancer stage where we found that LUAD, THCA, KIRP, and KIRC experienced a progression in the tumor stage with CHD1L expression. Not only tumor stage but also tumor metastasis showed a positive correlation with CHD1L expression in colon, liver, lung, and skin cancers. Our last differential comparison was based on the CHD1L protein levels analysis in normal and tumor tissues and again the trend of elevated CHD1L expression in tumor tissues was observed in LUAD, LIHC, glioblastoma multiforme, colon, and breast cancers where IHC staining, that was high for CHD1L in analyzed tumor tissues, confirmed our findings.

Survival analysis is a basic point of investigation for the assessment of disease progression and the patient’s response to medical treatment (Nagy et al., 2021). Consequently, the current study aimed to find the correlation between CHD1L expression and patients’ survival. The results from the GEPIA database demonstrated a positive correlation between CHD1L expression and the poor prognosis in ACC and SARC in terms of DFS and OS. Moreover, the output from KM plot analysis confirmed this positive correlation in all studied models of ovarian, gastric, and liver cancers which recommends the usage of CHD1L as a prognostic biomarker in the above-mentioned tumors. Several genes’ mutations were found to be a good prognostic marker for human cancer; examples include mutated *KRAS* that was correlated with poor prognosis of pancreatic (Buscail et al., 2020) and lung cancer (Shen et al., 2017) and mutated *NRAS* that was correlated with poor prognosis of metastatic melanoma (Jakob et al., 2012). Therefore, Our next step of the survival analysis was to study if the CHD1L genetic alteration could also affect patients’ survival where we found that CHD1L genetic alteration reflected a poor prognosis in terms of disease-free survival.

The status of gene methylation has been extensively studied in several human cancers. Previous studies generally found that DNA hypermethylation was a major mechanism for the silencing of tumor suppressor genes (Anglim et al., 2008). On the other hand, oncogenes experienced a hypomethylation status as a mechanism for their activation to induce tumor progression (Romero-Garcia et al., 2020); for example, a hypomethylation state was reported for the oncogenes *AQP1*, *LINE-1*, and *ELMO3* in salivary gland adenoid cystic carcinoma (Shao et al., 2011), colorectal cancer (Hur et al., 2014), and lung cancer (Søes et al., 2014) respectively. From this point, we performed a methylation analysis for CHD1L, and as expected several tumors including UCEC, COAD, PRAD, BLCA, LIHC, HNSC, TGCT, BRCA, and THCA showed hypomethylation in tumor samples versus the normal one. Additionally, CpG aggregated methylation data revealed that all of the significant results were in favor of CHD1L hypomethylation in the tumor sample versus normal one (except for CHOL).

Tumor immunotherapy, which witnessed a great evolution in the last few decades, became a well-established approach for fighting against cancer (Peng et al., 2019) where immune checkpoint inhibitors such as αPD-1 have been approved for the treatment of many types of human cancer such as malignant melanoma, gastric carcinoma, and hepatocellular carcinoma (Chang et al., 2021). For this purpose, it was important to study the correlation between elevated CHD1L expression in tumor tissue and the tumor infiltration of different types of immune cells. The first analyzed cell was MDSC, which was found to be positively affecting tumor cell survival and metastasis (Condamine et al., 2015). Additionally, it inhibits other cells with fighting ability against growing tumors (CD8 T cells and NK cells), supports tumor angiogenesis, and is involved in the formation of cancer stem cells (Weber et al., 2018). Consequently, it was not surprising that the elevated level of MDSC infiltration was correlated with poor clinical outcome for cancer patients (Zhang et al., 2016). The current study revealed that ACC, BLCA, BRCA, CESC, COAD, HNSC, HNSC-HPV-, HNSC-HPV+, KIRP, LIHC, LUAD, LUSC, OV, READ, SARC, SKCM, STAD, TGCT, UCEC, and UCS experienced a positive correlation between CHD1L expression and MDSC infiltration. It is noteworthy that cytokines such as CCL2, CCL5, and CSF1 were found to be involved in the attraction of MDSCs to the tumor site (Kumar et al., 2016). However, our finding of the positive association between CHD1L expression and MDSC infiltration is not fully investigated yet where the correlation between CHD1L upregulation and specific chemokine expression could be a possible mechanism that might explain this correlation. The second cell that was investigated for its correlation with CHD1L upregulation is NKT cell. This kind of cell demonstrated important roles in fighting against early tumors where it participates in cancer immune surveillance and secretes several effector molecules (Bae et al., 2019). Due to its tumor suppressive roles, NKT cell abundance in the tumor tissue was found to be a positive prognostic factor for patients’ survival in several human cancers (Wolf et al., 2018). Our results revealed that a significant negative correlation between CHD1L expression and NKT infiltration was found in BLCA, BRCA, CESC, COAD, HNSC, HNSC-HPV-, KIRC, KIRP, LUAD, MEOV, PRAD, SKCM, STAD, THYM, and UVM. Another interesting finding is that not even one tumor in our analyzed list experienced a positive between CHD1L expression and NKT infiltration. Putting the results of CHD1L expression and both MDSC and NKT infiltration together we can conclude that the upregulation of CHD1L expression could be used as a marker for a poor immune response against a growing tumor.

MSI and TMB are considered promising biomarkers for the patient’s response to immunotherapy (Zhao et al., 2020), where a robust antitumor effect of αPD1 treatment was observed with colorectal cancer patients with high microsatellite instability (MSI-H) (Diaz et al., 2017). Similarly, high TMB was positively correlated with a better clinical outcome across diverse tumors (Goodman et al., 2017). For this purpose, we tried to find if there is a correlation between the upregulation of CHD1L in tumor tissue and those promising biomarkers and our analysis revealed a positive correlation between MSI in READ, LUSC, UCEC, and BRCA and CHD1L expression. Additionally, ACC, PRAD, TGCT, LIHC, and READ experienced a positive correlation between CHD1L expression and TMB level. Collectively, our findings exposed a research question about the probability of relying on CHD1L expression in the above-mentioned tumor as a potential biomarker for patients’ response to tumor immunotherapy.

As a final point of analysis, the current study aimed to investigate the molecular mechanism of CHD1L in tumor progression where the top 50 interacting proteins and top 100 correlated proteins to CHD1L in the tumor tissue were obtained from STRING and GEPIA2 databases respectively and we interestingly found that PARP1 was a common protein in both of the generated datasets. This protein was found to be a regulator for prostate cancer growth and progression through transcriptional regulatory functions (Schiewer et al., 2012), also it was highly expressed in SCLC where its knockdown lead to SCLC growth inhibition (Byers et al., 2012). Moreover, PARP1 was found to be a prognostic biomarker for a poor clinical outcome in breast cancer patients (Mazzotta et al., 2016), therefore PARP1 inhibitors were extensively studied as a promising class of anticancer agents (Liang and Tan, 2010). Analysis of CHD1L correlated proteins in the tumor tissue revealed that POLR3C, PRKAB2, SETDB1, GPATCH4, and MSTO1 were the top five ones. SETDB1 was implicated as an oncogene in several human tumors (Lazaro-Camp et al., 2021) where it was involved in tumor progression in HCC through the methylation of p53 (Fei et al., 2015). Furthermore, SETDB1 has been involved in NSCLC progression through WNT–β-catenin pathway stimulation, and for these roles, it was nominated to be a therapeutic target to fight against numerous cancers (Sun et al., 2015). It is noteworthy that the detailed oncogenic roles of POLR3C, PRKAB2, SETDB1, and GPATCH4 have not been studied yet and because of being from the top correlating proteins with CHD1L in tumor tissues, their potential oncogenic roles, and interacting network should be further studied to present clues for novel tumor treatment strategies. Other points of assessment such as histone acetylation as a regulatory mechanism for CHD1L expression in cancerous tissue, the roles of non-coding RNA in controlling CHD1L and consequently affecting the tumor progression, and the single nucleotide polymorphism potential effect on the functions of CHD1L are important research points that should be investigated in a future work.

## 5 Conclusion

CHD1L is an oncogene that was found to be highly expressed as mRNA and protein in several human tumors and its upregulation was correlated with poor clinical outcomes. It affects the infiltration of several immune cells where immunosuppressive cells (MDSC) infiltration was positively correlated with CHD1L expression while the infiltration of tumor-fighting cells (NKT cells) was negatively correlated with the same gene. Also, TMB and MSI were found to be correlated with CHD1L expression in some human cancers therefore these findings could nominate CHD1L as a prognostic biomarker, a marker for patients’ response to immunotherapy, and a potential target for cancer treatment.

## Data Availability

The original contributions presented in the study are included in the article/Supplementary Material, further inquiries can be directed to the corresponding authors.
