# Supplementary material for: A pan-cancer analysis reveals CHD1L as a prognostic and immunological biomarker in several human cancers
Source: Front Mol Biosci. 2023 Mar 23;10:1017148. doi: 10.3389/fmolb.2023.1017148 (PMC10076660; doi:10.3389/fmolb.2023.1017148)
Supplement: Supplementary file 1 [file Table1.DOCX]

**A Pan-Cancer Analysis Reveals CHD1L as a Prognostic and Immunological Biomarker in Several Human Cancers**

Mohamed A. Soltan*+^1^, Muhammad Alaa Eldeen+^2^, Refaat A. Eid^3^, Najiah M. Alyamani^4^, Leena S. Alqahtani^5^, Sarah Albogami^6^, Ibrahim Jafri^6^, Moon Nyeo Park^7^, Ghadi Alsharif^8^, Eman Fayad^6^, Gamal Mohamed^9^, Rihab Osman^10^, Bonglee Kim^7,*#^, & Mohamed Samir A. Zaki^11&12#^

**Supplementary material**

**1) Supplementary figures**


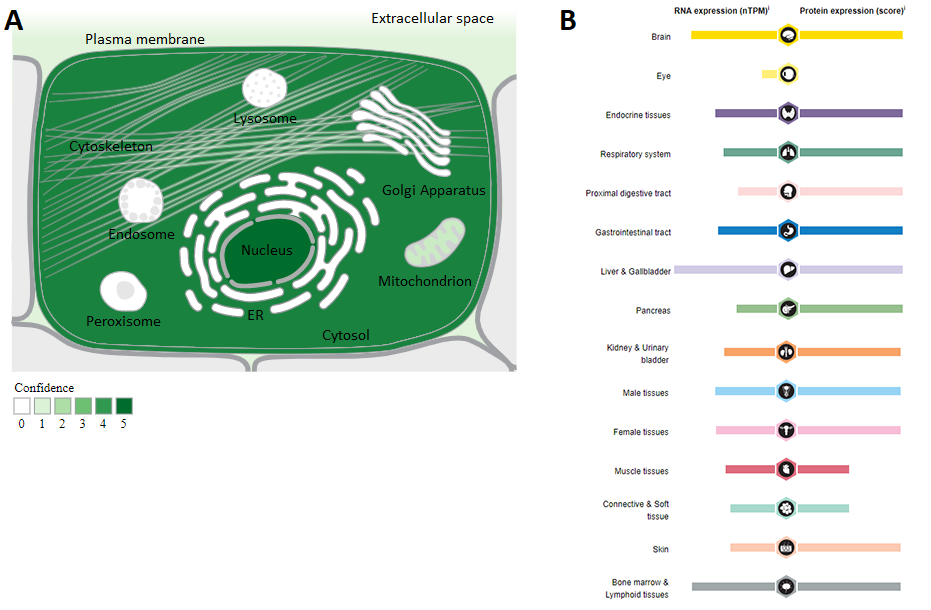


Supplementary figure1. A) Subcellular localization of CHD1L. B) Tissue distribution of CHD1L as a protein and RNA.


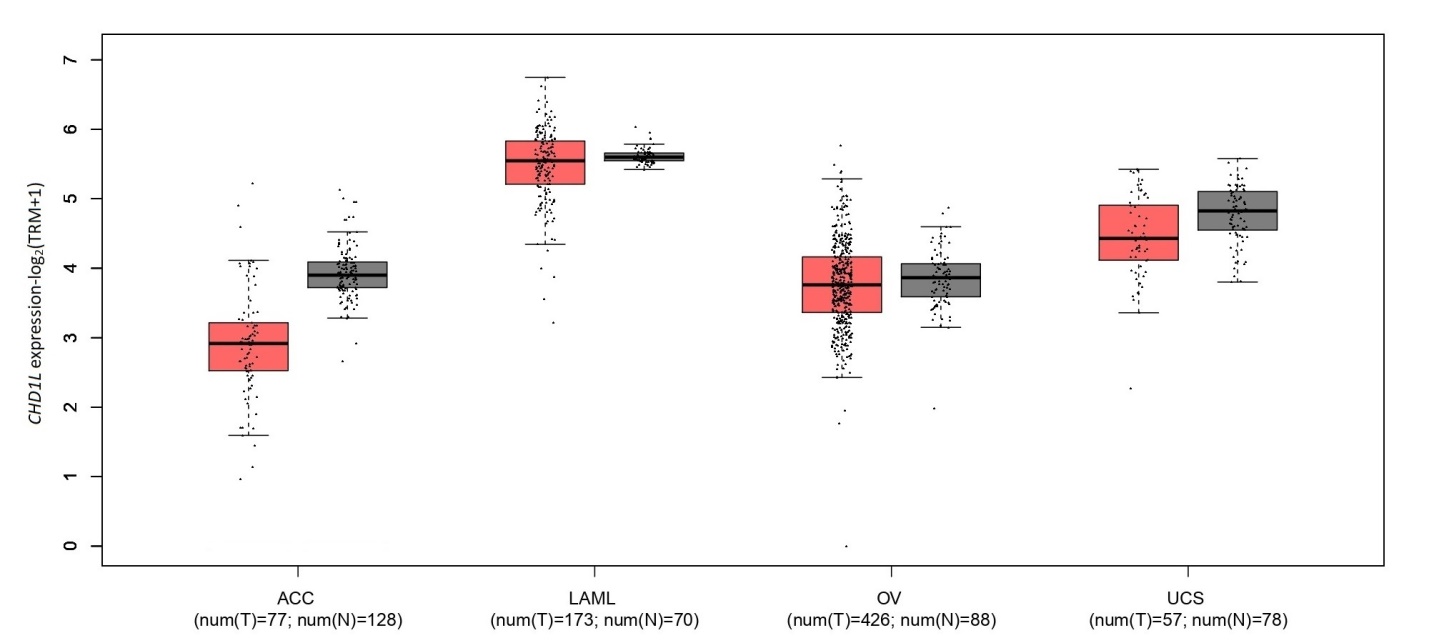


Supplementary figure2. The tumors that lack normal tissue for comparison in TIMER2.0. database and experienced nonsignificant CHD1L expression variation in tumor versus normal tissue when analyzed in the GEPIA database.


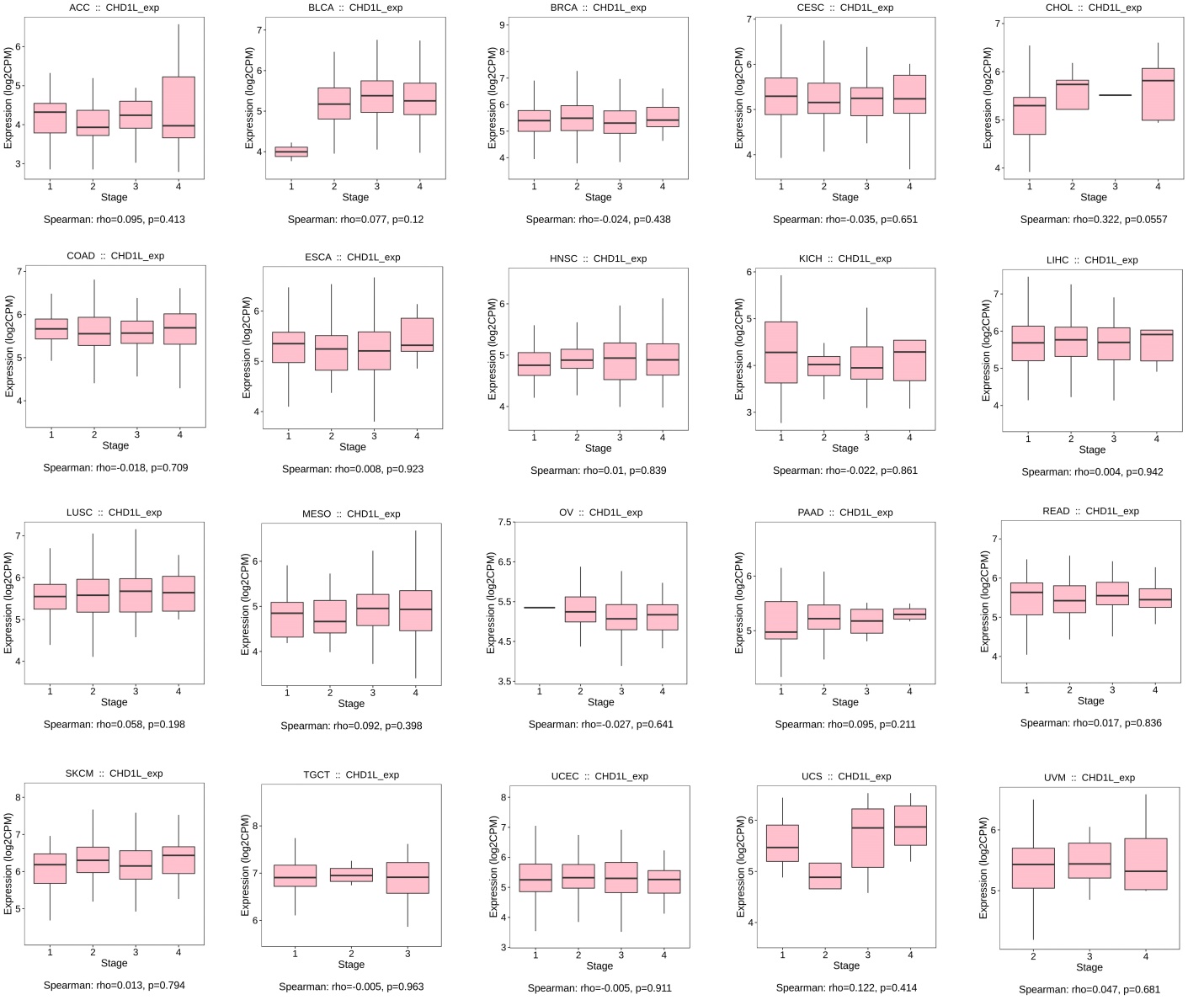


Supplementary figure 3. Tumors experienced a nonsignificant correlation between CHD1L expression and tumor stage.


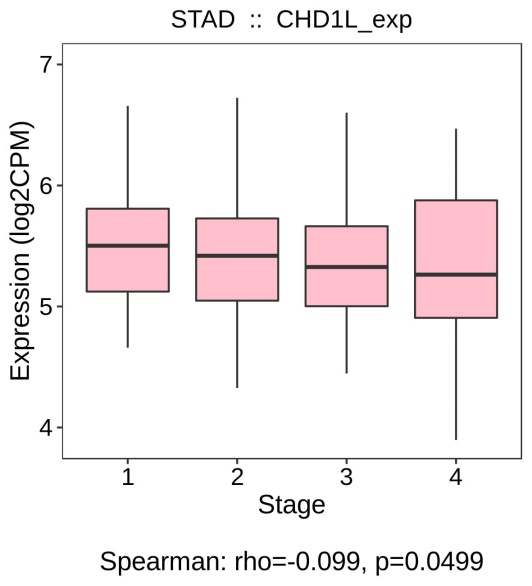


Supplementary figure 4. STAD experienced a significant negative correlation between CHD1L expression and tumor stage.


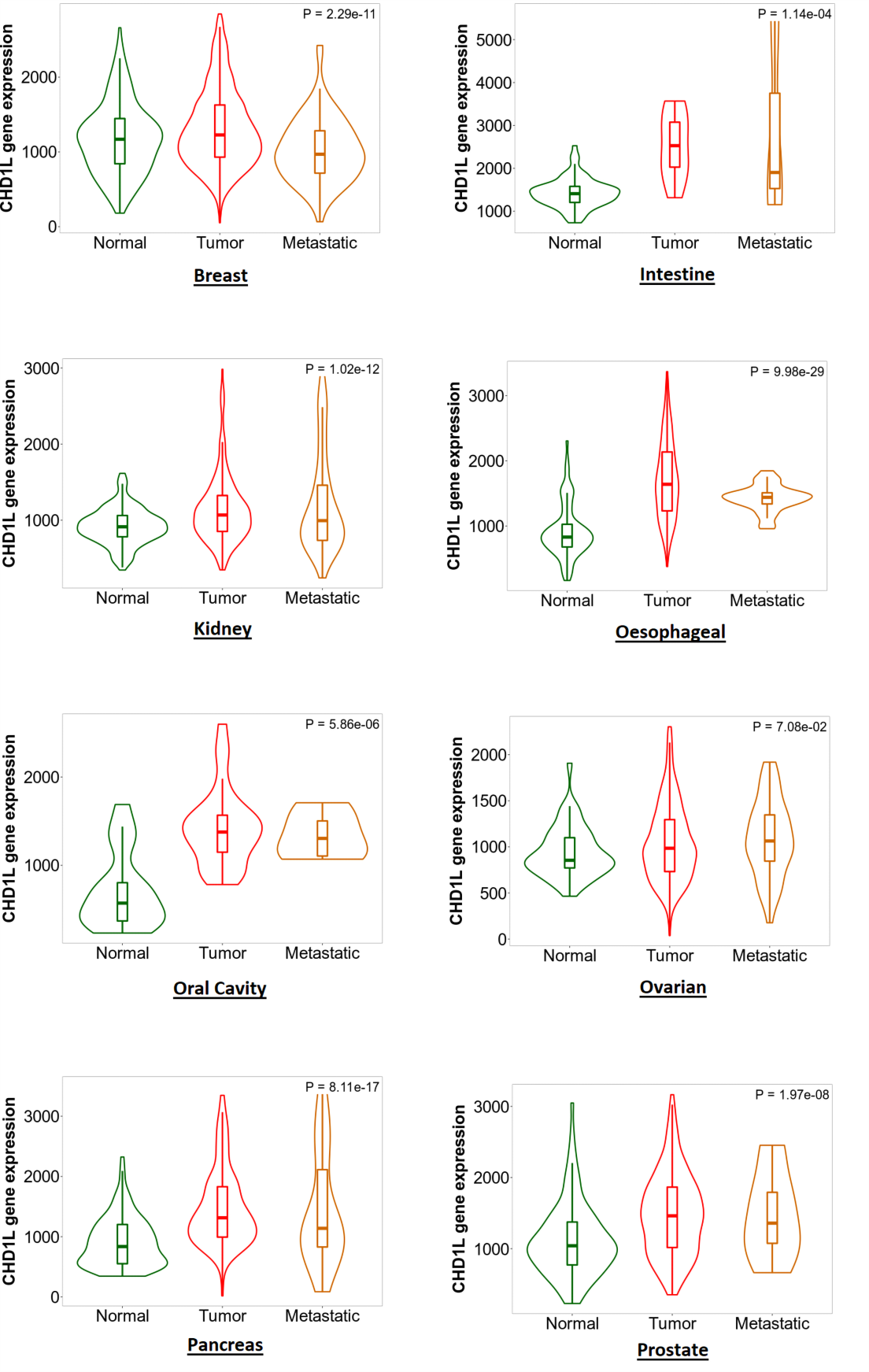


Supplementary figure 5. Tumors experienced a non-continuous positive correlation between CHD1L expression and tissue type (normal-tumor-metastatic).


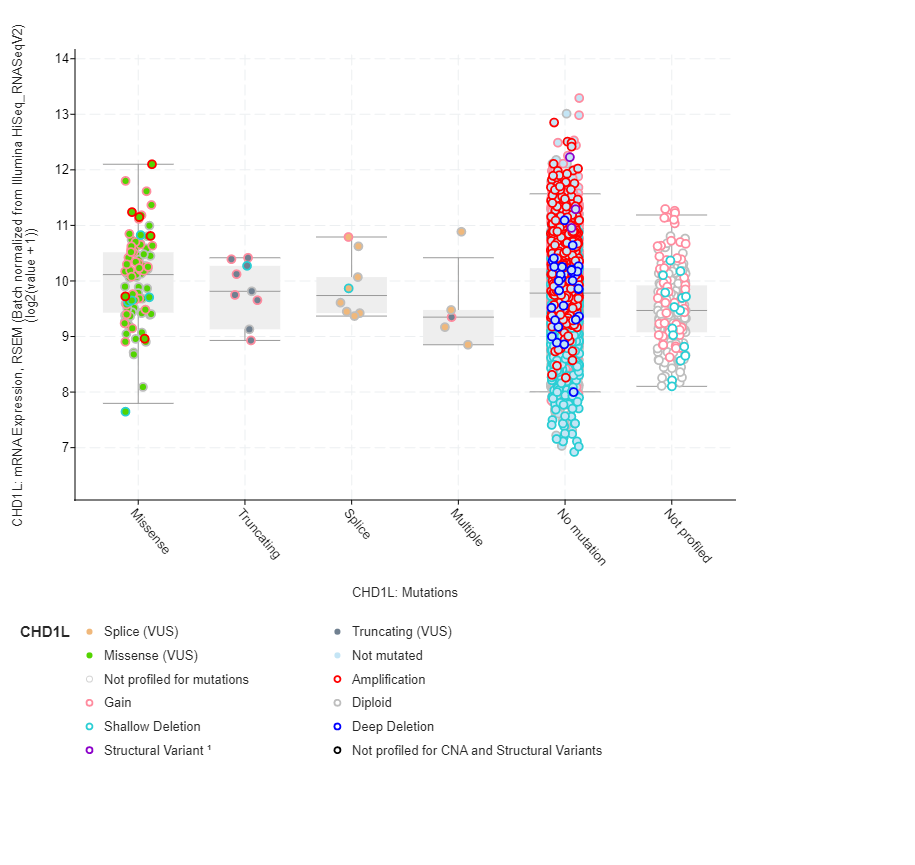


Supplementary figure 6. The correlation between CHD1L expression and mutation types as assessed by the cBioPortal web server.


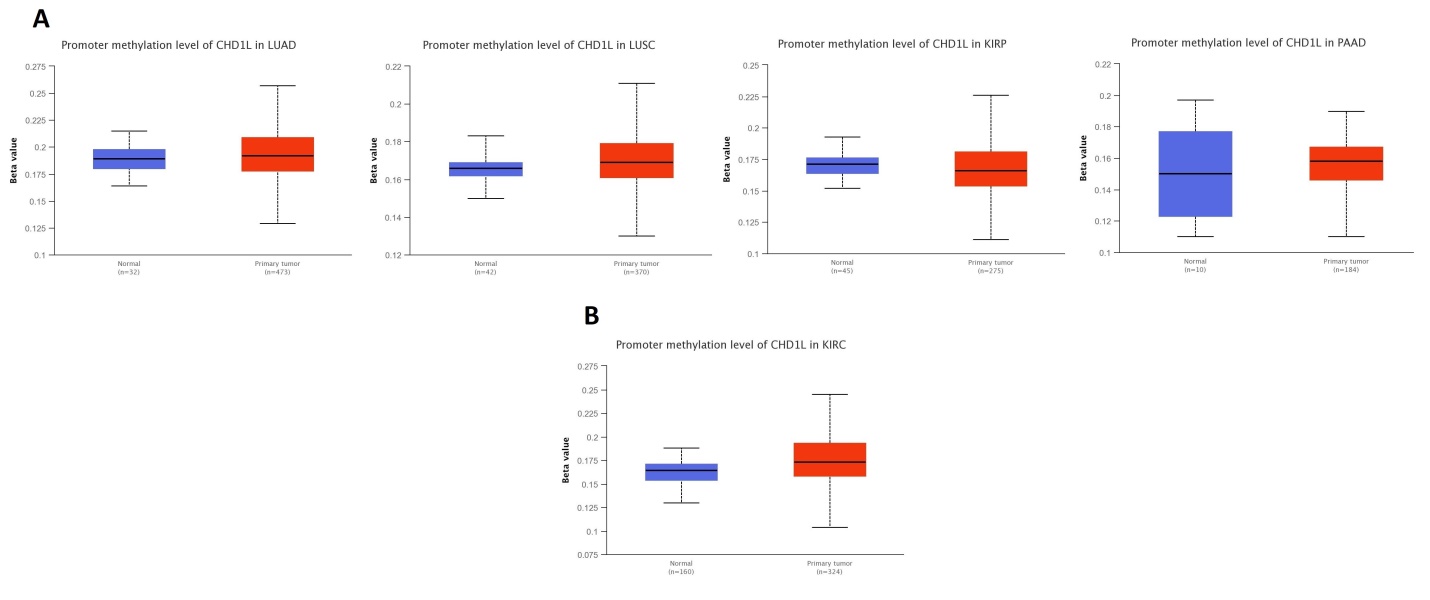


Supplementary figure 7. Differential methylation analysis of CHD1L in tumor samples versus normal ones. (A) Tumors experienced a nonsignificant difference in the methylation level of CHD1L promoter in tumor and normal samples. (B) Tumor experienced significantly lower promoter methylation in normal versus tumor samples.


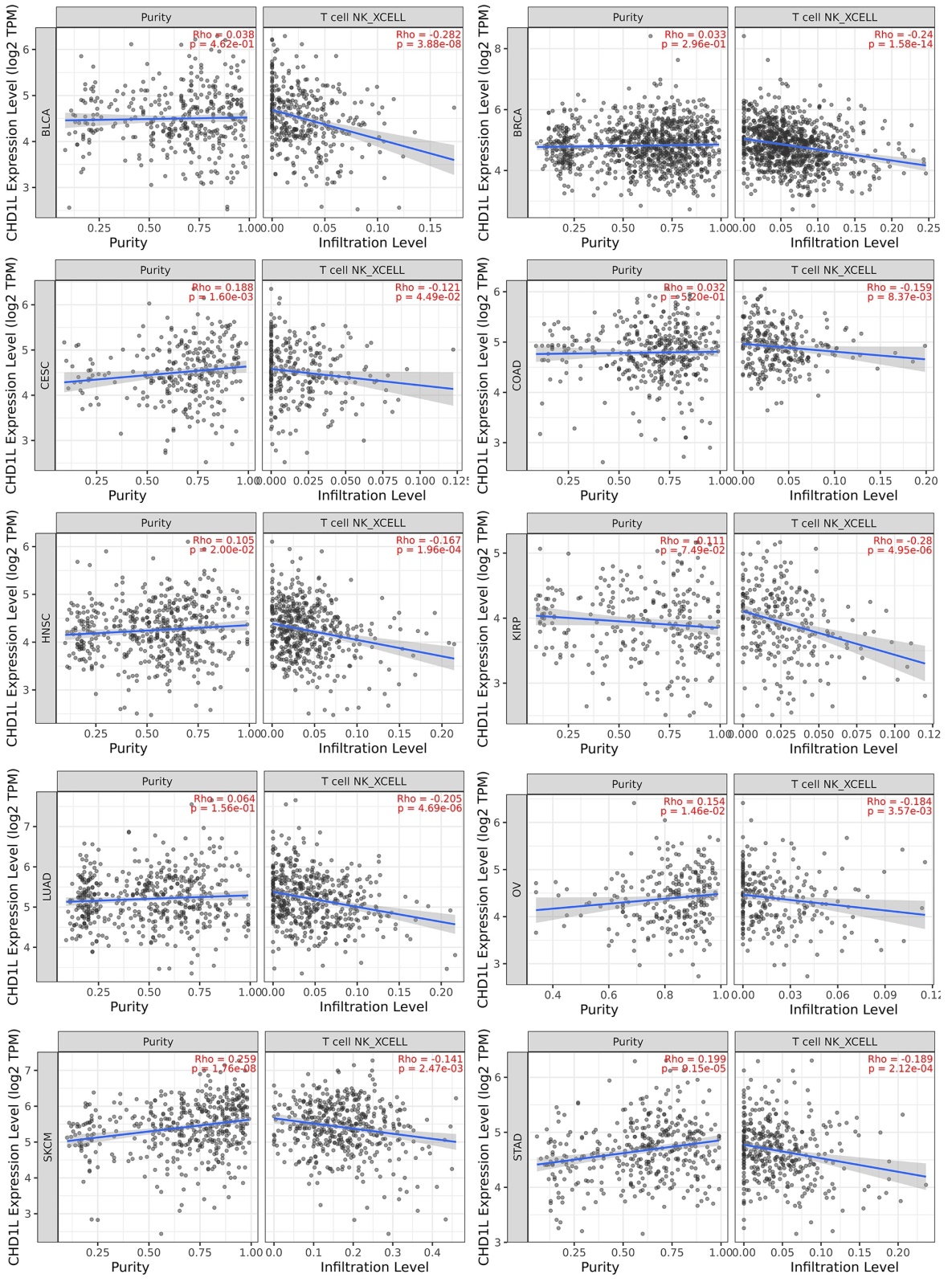


Supplementary figure 8. Scatter plots that demonstrate the correlation between the expression of CHD1L and the infiltration level of natural killer T cells.

**1) Supplementary tables**

Supplementary Table 1. The abbreviations and the full name of analyzed tumors in the current study

| Abbrviation | Tumor name |
| --- | --- |
| ACC | Adrenocortical carcinoma |
| BLCA | Bladder Urothelial Carcinoma |
| BRCA | Breast invasive carcinoma |
| CESC | Cervical squamous cell carcinoma and endocervical adenocarcinoma |
| CHOL | Cholangiocarcinoma |
| COAD | Colon adenocarcinoma |
| DLBC | Lymphoid Neoplasm Diffuse Large B-cell Lymphoma |
| ESCA | Esophageal carcinoma |
| GBM | Glioblastoma multiforme |
| HNSC | Head and Neck squamous cell carcinoma |
| KICH | Kidney Chromophobe |
| KIRC | Kidney renal clear cell carcinoma |
| KIRP | Kidney renal papillary cell carcinoma |
| LAML | Acute Myeloid Leukemia |
| LGG | Brain Lower Grade Glioma |
| LIHC | Liver hepatocellular carcinoma |
| LUAD | Lung adenocarcinoma |
| LUSC | Lung squamous cell carcinoma |
| MESO | Mesothelioma |
| OV | Ovarian serous cystadenocarcinoma |
| PAAD | Pancreatic adenocarcinoma |
| PCPG | Pheochromocytoma and Paraganglioma |
| PRAD | Prostate adenocarcinoma |
| READ | Rectum adenocarcinoma |
| SARC | Sarcoma |
| SKCM | Skin Cutaneous Melanoma |
| STAD | Stomach adenocarcinoma |
| TGCT | Testicular Germ Cell Tumors |
| THCA | Thyroid carcinoma |
| THYM | Thymoma |
| UCEC | Uterine Corpus Endometrial Carcinoma |
| UCS | Uterine Carcinosarcoma |
| UVM | Uveal Melanoma |
